# Supplementary material for: Comparative genomics provides new insights into the diversity, physiology, and sexuality of the only industrially exploited tremellomycete: Phaffia rhodozyma
Source: BMC Genomics. 2016 Nov 9;17:901. doi: 10.1186/s12864-016-3244-7 (PMC5103461; doi:10.1186/s12864-016-3244-7)
Supplement: Additional file 6: — List of orphan genes with links to PFAM (related to Additional file 1: Table S1). (ZIP 1428 kb) [file 12864_2016_3244_MOESM6_ESM.zip › BLAST_HTML_FTR/G00232_P.html]

BLAST Search Results


```
BLASTP 2.2.27+


Reference:
Stephen F. Altschul, Thomas L. Madden, Alejandro A. Schäffer,
Jinghui Zhang, Zheng Zhang, Webb Miller, and David J. Lipman (1997),
"Gapped BLAST and PSI-BLAST: a new generation of protein database
search programs", Nucleic Acids Res. 25:3389-3402.


Reference for
composition-based statistics:
Alejandro A. Schäffer, L. Aravind, Thomas L. Madden, Sergei
Shavirin, John L. Spouge, Yuri I. Wolf, Eugene V. Koonin, and
Stephen F. Altschul (2001), "Improving the accuracy of PSI-BLAST
protein database searches with composition-based statistics and
other refinements", Nucleic Acids Res. 29:2994-3005.


Database: nr
           71,551,133 sequences; 26,053,659,533 total letters


Query= G00232_P

Length=217
                                                                      Score     E
Sequences producing significant alignments:                          (Bits)  Value

emb|CDZ98309.1|  hypothetical protein [Xanthophyllomyces dendrorh...   421    3e-147
gb|KIJ17561.1|  hypothetical protein PAXINDRAFT_9506 [Paxillus in...  41.2    0.40  
ref|WP_013108804.1|  hypothetical protein [Planctopirus limnophil...  40.0    1.4   
gb|KIK57406.1|  hypothetical protein GYMLUDRAFT_263155 [Gymnopus ...  39.3    1.6   
ref|XP_007866942.1|  hypothetical protein GLOTRDRAFT_139203 [Gloe...  38.1    3.3   
ref|XP_002583349.1|  conserved hypothetical protein [Uncinocarpus...  38.9    3.5   
ref|XP_012182852.1|  predicted protein [Fibroporia radiculosa] >e...  37.0    4.0   
ref|WP_048495036.1|  chemotaxis protein CheY [Marinobacter subter...  37.4    8.5   
gb|EUC58717.1|  hypothetical protein RSOL_273320 [Rhizoctonia sol...  37.4    8.5   


 >emb|CDZ98309.1| hypothetical protein [Xanthophyllomyces dendrorhous]
Length=216

 Score =  421 bits (1083),  Expect = 3e-147, Method: Compositional matrix adjust.
 Identities = 216/216 (100%), Positives = 216/216 (100%), Gaps = 0/216 (0%)

Query  1    MSAPSAIAVTDAPAAHLGRPLTSSDTISAGIPDSIVTSQTTTGHVHDATNTATGNNTGKA  60
            MSAPSAIAVTDAPAAHLGRPLTSSDTISAGIPDSIVTSQTTTGHVHDATNTATGNNTGKA
Sbjct  1    MSAPSAIAVTDAPAAHLGRPLTSSDTISAGIPDSIVTSQTTTGHVHDATNTATGNNTGKA  60

Query  61   PIGDKISGEVDKLTGKAQKNPELVAQGVEKKGHADEAEAVRATQNTDTSRAAKETGAGDS  120
            PIGDKISGEVDKLTGKAQKNPELVAQGVEKKGHADEAEAVRATQNTDTSRAAKETGAGDS
Sbjct  61   PIGDKISGEVDKLTGKAQKNPELVAQGVEKKGHADEAEAVRATQNTDTSRAAKETGAGDS  120

Query  121  TDVKHTNVISPTSAGASHSAVSSVDPTLNNIGKAPIGQKISGEINRLVGKLTGNVEKVAQ  180
            TDVKHTNVISPTSAGASHSAVSSVDPTLNNIGKAPIGQKISGEINRLVGKLTGNVEKVAQ
Sbjct  121  TDVKHTNVISPTSAGASHSAVSSVDPTLNNIGKAPIGQKISGEINRLVGKLTGNVEKVAQ  180

Query  181  GTEQKGHAAKAQAARDLNTVDVSKAAKETGASDSKA  216
            GTEQKGHAAKAQAARDLNTVDVSKAAKETGASDSKA
Sbjct  181  GTEQKGHAAKAQAARDLNTVDVSKAAKETGASDSKA  216


>gb|KIJ17561.1| hypothetical protein PAXINDRAFT_9506 [Paxillus involutus ATCC 
200175]
Length=220

 Score = 41.2 bits (95),  Expect = 0.40, Method: Compositional matrix adjust.
 Identities = 23/76 (30%), Positives = 38/76 (50%), Gaps = 7/76 (9%)

Query  23   SSDTISAGIPDSIVTSQTTTGHVHD-------ATNTATGNNTGKAPIGDKISGEVDKLTG  75
            S DT    +P +      + G+  D       + N ATG   GK  +GD++ G V+K+ G
Sbjct  133  SDDTQDCNVPGAGRLGGESRGYERDNDEYDPSSDNFATGTQGGKPSLGDRMKGGVEKMAG  192

Query  76   KAQKNPELVAQGVEKK  91
            K  ++P L  +G ++K
Sbjct  193  KVMRDPHLEVKGEQRK  208


>ref|WP_013108804.1| hypothetical protein [Planctopirus limnophila]
 gb|ADG66373.1| protein of unknown function DUF1549 [Planctopirus limnophila 
DSM 3776]
Length=893

 Score = 40.0 bits (92),  Expect = 1.4, Method: Composition-based stats.
 Identities = 32/104 (31%), Positives = 50/104 (48%), Gaps = 15/104 (14%)

Query  13   PAAHLGRPLTSSDTISAGIPDSIVTSQTTTGH--------VHDATNTATGNNTGKAPIGD  64
            PAA L  PL S   +S   P  + T   TT H        ++ + + AT    G+ P+  
Sbjct  239  PAAKLDSPLASHSDLSTSDPAGLQTKGDTTAHPAAEINGPLNRSNSEATARTNGRTPVAL  298

Query  65   KISGEVDKLTGKAQKNP--ELVAQGV----EKKGHADEAEAVRA  102
             +S  +DK  G +Q NP  EL+AQ      E++  A+EA  +++
Sbjct  299  PLS-TLDKPQGVSQANPTEELIAQRAQALKEQQAMAEEAARIKS  341


>gb|KIK57406.1| hypothetical protein GYMLUDRAFT_263155 [Gymnopus luxurians FD-317 
M1]
Length=253

 Score = 39.3 bits (90),  Expect = 1.6, Method: Compositional matrix adjust.
 Identities = 21/55 (38%), Positives = 34/55 (62%), Gaps = 2/55 (4%)

Query  62   IGDKISGEVDKLTGKAQKNPELVAQGVEKKGHADEAEAVRATQNTDTSRAAKETG  116
            +GDK++G  ++L GK  KNPELV QG E++    E +  +  ++T+ +   KE G
Sbjct  103  VGDKLTGLKEELKGKVTKNPELVEQGKERR--TGELKKKQEQEDTEKNDPFKEPG  155


>ref|XP_007866942.1| hypothetical protein GLOTRDRAFT_139203 [Gloeophyllum trabeum 
ATCC 11539]
 gb|EPQ54675.1| hypothetical protein GLOTRDRAFT_139203 [Gloeophyllum trabeum 
ATCC 11539]
Length=193

 Score = 38.1 bits (87),  Expect = 3.3, Method: Compositional matrix adjust.
 Identities = 17/37 (46%), Positives = 26/37 (70%), Gaps = 1/37 (3%)

Query  56   NTGKAP-IGDKISGEVDKLTGKAQKNPELVAQGVEKK  91
            NTGK P  GDK+ G ++K+ GK  +NP +V +G ++K
Sbjct  152  NTGKKPGFGDKLMGGLEKVAGKTTRNPGMVERGEDRK  188


>ref|XP_002583349.1| conserved hypothetical protein [Uncinocarpus reesii 1704]
 gb|EEP81451.1| conserved hypothetical protein [Uncinocarpus reesii 1704]
Length=605

 Score = 38.9 bits (89),  Expect = 3.5, Method: Compositional matrix adjust.
 Identities = 39/141 (28%), Positives = 61/141 (43%), Gaps = 31/141 (22%)

Query  47   DATNTATGNNTGKAPIGDKISGEVDKLTGKAQKNPELVAQGVEKKGHADEAEAVRATQNT  106
            DA    +G + G+  I D  SG++     +    P   A+ V +    +E   + AT++ 
Sbjct  475  DANRVVSGGSDGRVKIWDLASGQL----VRELSQP---AEAVWRVAFEEEKAVILATRD-  526

Query  107  DTSRAAKETGAG-----DSTDVKHTNVISPTS------AGASHSAVSSVDPTLNNIG--K  153
               R   E  +      D  +  HT  +SPTS        A+HS +++VD T +N+G   
Sbjct  527  --GRTLMEVWSFSPPEDDYAETYHTGSVSPTSTLGQSSFSAAHSTITTVDATSSNVGTVS  584

Query  154  APIG--------QKISGEINR  166
            +PI         Q IS E NR
Sbjct  585  SPISYELHDLEMQDISNESNR  605


>ref|XP_012182852.1| predicted protein [Fibroporia radiculosa]
 emb|CCM03569.1| predicted protein [Fibroporia radiculosa]
Length=125

 Score = 37.0 bits (84),  Expect = 4.0, Method: Compositional matrix adjust.
 Identities = 19/34 (56%), Positives = 23/34 (68%), Gaps = 0/34 (0%)

Query  152  GKAPIGQKISGEINRLVGKLTGNVEKVAQGTEQK  185
            GK  IGQKI GE+  + GKLT N  KV +G E+K
Sbjct  89   GKPSIGQKIKGELEEVAGKLTRNPGKVERGKEEK  122


>ref|WP_048495036.1| chemotaxis protein CheY [Marinobacter subterrani]
 gb|KMQ74871.1| Response regulator receiver domain [Marinobacter subterrani]
Length=364

 Score = 37.4 bits (85),  Expect = 8.5, Method: Compositional matrix adjust.
 Identities = 35/125 (28%), Positives = 58/125 (46%), Gaps = 5/125 (4%)

Query  28   SAGIPDSIVTSQTTTGHVHDATNTATGNNTGKAPIGDKISGEVDKLTGKAQKN--PELVA  85
            +AGIP  + TS+ ++  + +A N    N   K P  D +S  +D+L     +   PE +A
Sbjct  76   TAGIPIIMCTSKKSSSFMEEARNFGVYNILTKPPQTDGLSQVLDQLAIDVTRGSLPEPLA  135

Query  86   QGVEKKGHADEAEAVRATQNTDTSRAAKETGAGDSTDVKHTNVISPTSAGASHSAVSSVD  145
               +K  H ++ E +       + + + +TG GDS      NV+  TS      A S+V 
Sbjct  136  ---DKAAHPEDEEDLLNIPADASVQLSPKTGDGDSRGGPQANVVPLTSELIEQIARSAVK  192

Query  146  PTLNN  150
              +NN
Sbjct  193  THINN  197


>gb|EUC58717.1| hypothetical protein RSOL_273320 [Rhizoctonia solani AG-3 Rhs1AP]
 gb|KEP51354.1| hypothetical protein V565_063230 [Rhizoctonia solani 123E]
Length=269

 Score = 37.4 bits (85),  Expect = 8.5, Method: Compositional matrix adjust.
 Identities = 14/33 (42%), Positives = 23/33 (70%), Gaps = 0/33 (0%)

Query  59   KAPIGDKISGEVDKLTGKAQKNPELVAQGVEKK  91
            K  +G+K+ G ++ + GK  KNPEL+ +G E+K
Sbjct  230  KPTLGEKVKGTMETIRGKVTKNPELIERGQERK  262


Lambda      K        H        a         alpha
   0.305    0.121    0.323    0.792     4.96 

Gapped
Lambda      K        H        a         alpha    sigma
   0.267   0.0410    0.140     1.90     42.6     43.6 

Effective search space used: 1149771635813


  Database: nr
    Posted date:  Sep 23, 2015 12:05 AM
  Number of letters in database: 26,053,659,533
  Number of sequences in database:  71,551,133


Matrix: BLOSUM62
Gap Penalties: Existence: 11, Extension: 1
Neighboring words threshold: 11
Window for multiple hits: 40
```
